# Complete mitochondrial genome of the Asian badger *Meles leucurus* (Mustelidae) from Korea

**DOI:** 10.1080/23802359.2017.1365648

**Published:** 2017-08-17

**Authors:** Mi Gyeong Jeon, Hey Ri Kim, Ji Hong Min, Hyun Ju Kim, Yung Chul Park

**Affiliations:** a Ecosystem Research Division, National Park Research Institute, Korea National Park Service, Wonju, Republic of Korea;; b Division of Forest Science, College of Forest & Environmental Sciences, Kangwon National University, Chuncheon, Republic of Korea;; c Microbial Safety Team, National Institute of Agricultural Sciences, Rural Development Administration, Wanju, Republic of Korea

**Keywords:** Mitogenome, Asian badger, *Meles leucurus*, Mustelidae

## Abstract

The mitogenome of the Asian badger *Meles leucurus* from Korea is a circular molecule of 16,529 bp, consisting of a control region and a conserved set of 37 genes containing 13 protein-coding genes (PCGs), 22 tRNA genes, and two rRNA genes (*12S rRNA* and *16S rRNA*). The mitogenome of *M. leucurus* is AT-biased, with a nucleotide composition of 33.1% A, 27.9% T, 25.4% C, and 13.5% G. The phylogenetic analysis revealed that the badger *M. leucurus* from Korea is well grouped with that from China, forming a sister clade to *M. meles* from Japan.

The Asian badger *Meles leucurus*, also known as the sand badger, is found in Mongolia, China, Kazakhstan, the Korean Peninsula, and Russia (Abramov 2016). We sequenced and characterized the complete mitogenome of the Asian badger *M. leucurus* (Mustelidae) from Korea. Genomic DNA was extracted from a road-killed individual around agroecosystem in Odaesan National Park (N37 45 26.4, E128 36 39.8). The voucher specimen (MUMELE-1) was deposited in the National Park Research Institute, Korea National Park Service. Genomic DNA extraction, PCR, and gene annotation were conducted according to the previous studies (Yoon et al. 2013; Jeon and Park 2015; Rahman et al. 2016). Previously published mitogenomes of the Eurasian *M. meles* (AM711900) (Arnason et al. 2007) and Japanese *M. anakuma* (AB291075) (Yonezawa et al. 2007) were used as references for gene annotation and primer design for PCR amplification of the Korean *M. leucurus*. Phylogenetic tree was constructed using maximum likelihood (ML) procedures implemented in MEGA6 (Tamura et al. 2013).

The complete mitogenome (MF497304) of *M. leucurus* is 16,529 bp total length, which consists of a CR and a conserved set of 37 vertebrate mitochondrial genes including 13 protein-coding genes (PCGs), 22 tRNA genes, and two rRNA genes (*12S rRNA* and *16S rRNA*). The order and orientation of these genes are identical to those other mammalian species (Kim and Park 2012; Yoon et al. 2013; Nam et al. 2015). The mitogenome of *M. leucurus* is AT-biased, with a nucleotide composition of 33.1% A, 27.9% T, 25.4% C, and 13.5% G.

Total length of 13 PCGs consists of 11,382 bp, with the exclusion of stop codons, which encode 3794 amino acids. The start codon ATG is used in all the other PCGs except for *Nd2* (ATT), *Nd3* (ATA), and *Nd5* (ATC). The stop codon TAA is used for termination of seven PCGs (*Cox1*, *Cox2*, *Atp8*, *Atp6*, *Nd4L*, *Nd5*, and *Nd6*), while AGA and TAG occur once in *Cytb* and *Nd3*, respectively. Incomplete stop codon T– is found in *Nd1*, *Nd2*, *Cox3*, and *Nd4*. Ribosomal RNA genes (*12srRNA* and *16srRNA*) are located between *tRNA^Phe^* and *tRNA^Leu(CUN)^* and separated by *tRNA^Val^*. Lengths of *12S rRNA* and *16S rRNA* gene were 960 bp and 1573 bp long, respectively. Length of 22 tRNA genes for transferring 20 amino acids ranges from 62 bp (*tRNA^Ser (AGY)^*) to 75 bp (*tRNA^Leu (UUR)^*). Lengths of the two-non coding genes are 1088 bp (control region) and 36 bp (*O_L_*) long, respectively.

The phylogenetic analysis revealed that the badger *M. leucurus* from Korea is well grouped with that from China, forming a sister clade to *M. meles* from Japan. The clade of the genus *Meles* was strongly supported with high bootstrap values and formed a sister clade to the hog badger *Arctonyx collaris* (Figure 1).

**Figure 1.**
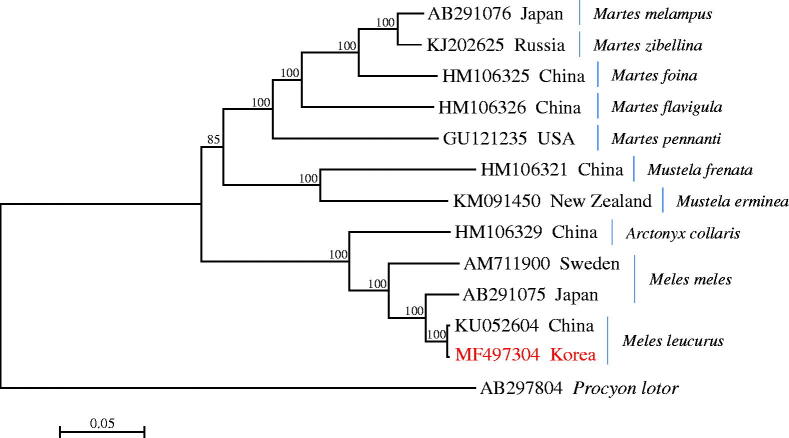
The phylogenetic relationship of the Asian badger *Meles leucurus* and its allied species inferred from maximum-likelihood analysis based on mitogenome sequences. The Korean badger *M. leucurus* used in this study was indicated with the mitogenome accession no. MF497304. The ML tree was generated using the GTR + G+I model, and the robustness of the tree was tested with 1000 bootstrap. The numbers on the branches indicate bootstrap values.
